# The Implications of Using Digital Technologies in the Management of COVID-19: Comparative Study of Japan and South Korea

**DOI:** 10.2196/45705

**Published:** 2023-06-06

**Authors:** Sou Hee Yang

**Affiliations:** 1 Faculty of Social Sciences Waseda University Tokyo Japan

**Keywords:** comparative study, technology, health care technology, digital technology, COVID-19, mobile phone

## Abstract

**Background:**

Technology can assist in providing effective infectious disease management, but it can also become a source of social injustice and inequality. To control the rapidly increasing SARS-CoV-2 infections and promote effective vaccine administration, both South Korea and Japan have been using several technology-based systems and mobile apps. However, their different approaches to technology use have yielded contrasting social implications.

**Objective:**

Through comparative studies of the use of digital technologies for pandemic management and its social implications in Japan and South Korea, this study aimed to discuss whether the active and optimal use of technology for pandemic management can occur without subverting or compromising important social values, such as privacy and equality.

**Methods:**

This study compared the social implications of Japan’s and South Korea’s contrasting approaches to technology implementation for COVID-19 pandemic management in early 2022.

**Results:**

Digital technologies have been actively and comprehensively used in South Korea, enabling effective COVID-19 management, but have raised serious concerns about privacy and social equality. In Japan, technologies have been more carefully implemented, thereby not causing similar social concerns, but their effectiveness in supporting COVID-19 regulations has been criticized.

**Conclusions:**

Potential social implications such as equality concerns, the balance between public interest and individual rights, and legal implications must be carefully assessed in conjunction with effective and optimal infectious disease control to achieve sustainable use of digital health technologies for infectious disease management in the future.

## Introduction

During the COVID-19 pandemic, technology has become indispensable for effective infectious disease control [1]. However, the use of pandemic management technology has also caused many substantial social problems. Around March 2022, when Omicron became the dominant novel SARS-CoV-2 variant in South Korea and Japan, both governments sought to use technology to monitor those who were infected and under quarantine and to manage vaccinations for residents and those entering the country. South Korea has used high-end technologies, including blockchain technologies, to use mobile apps to monitor those under quarantine and to administer vaccine passes in public places [2]. Japan sought to do the same; however, its technological intervention was comparatively more limited, and public participation was largely voluntary [3]. Given the differences in technology use for the COVID-19 pandemic between these 2 countries, the resulting criticisms also varied. In South Korea, where the use of apps was more strongly encouraged or mandated, questions about human rights, equality concerns for those who cannot use the technology well, and concerns about technology errors or failures were raised [4-6]. In Japan, although its voluntary encouragement for participation from the public has not caused the same questions to be raised, the efficacy of technology use has been criticized [7]. The different approaches for adopting pandemic technologies and their societal implications in Japan and South Korea suggest giving importance to pertinent future considerations, including the need for public education about the technologies and planning technology use in a way that minimizes human right invasions, to promote effective use of pandemic management technology.

## Methods

### Procedure

To compare the use of technology for pandemic management and its social implications in Japan and South Korea, a case-oriented comparative study, based on document review, was conducted. The comparison was made for the period from the end of 2021 to the beginning of 2022, during which the use was most active. Although digital health can be broadly defined as “the use of information and communications technologies in medicine and other health professions to manage illnesses and health risks and to promote wellness” [8], for the purposes of this study, the scope of digital health was narrowed to app-based technologies intended for public use in Japan and South Korea for COVID-19 management (Table 1). The purposes and the intended use of the apps were not the same in the 2 countries. Thus, although exact comparisons are not possible, this study compared the apps used by governments to (1) monitor SARS-CoV-2 infections and provide medical assistance, including contact tracing and border protection, and (2) present COVID-19 vaccination certificates.

The following two aspects of the social implications of using technologies were analyzed: (1) the efficiency of technology use and (2) potential human rights violations and infringement of social values. Efficiency refers to how well the government employees and the public were able to use the apps for their intended purposes, considering the user-friendliness of the technologies, technical difficulties, and use of the app by the public. Human rights concerns included infringements of the right to privacy and the right to make one’s own medical decisions and the release of personal information. Concerns related to social values included equality and social inclusion concerns.

The analysis of the efficiency of technology use was performed based on a review of government policy documents, news articles, and reports by government employees [7,9-11]. The documents were analyzed for characteristics of technology use, technological difficulties, and the resolution of difficulties. Government reports and press releases, court decisions, newspaper articles, and academic articles related to digital technology use in Japan and South Korea from the end of 2021 to the beginning of 2022 were analyzed for any legal interventions, events, or incidents that occurred in the process of technology use [3,4,10,12]. Furthermore, the documents were analyzed for concerns related to human rights violations and the infringement of social values caused by using COVID-19 pandemic technologies. Although personal anecdotes were not included to avoid potential bias, public opinions from newspapers and opinions of government officials and workers who had firsthand experience of adopting digital technology were included to ensure that this study captured broad aspects of the experiences associated with technology use in the 2 countries [2,10]. On the basis of the analysis from the document review, social implications for Japan and South Korea were compared, and important social considerations when applying technologies for infectious disease regulation were highlighted.

**Table 1:** Apps used in Japan and South Korea that were compared in this study.

Purposes of digital technologies used for COVID-19 management	Apps used in Japan	Apps used in South Korea
Monitor SARS-CoV-2 infection, including contact tracing and border protection	HER-SYS^a^ COCOA^b^ MY SOS (quarantine management and border control)	Self-Quarantine Safety Protection App (quarantine management and border control) Contact tracing QR codes
COVID-19 vaccination certification	COVID-19 Vaccination Certificate App	COOV^c^

^a^HER-SYS: Health Center Real-time Information-Sharing System on COVID-19.

^b^COCOA: COVID-19 Contact Confirming App.

^c^COOV: Corona Overcome.

### Ethics Approval

An ethics board review is not required for this study, as this study is primarily based on document review.

## Results

### Japan’s Use of Digital Technology During the COVID-19 Pandemic

The Japanese government encouraged the public to use various apps and digital technologies to enable effective COVID-19 management, with some being more actively used than others. Health Center Real-time Information-Sharing System on COVID-19 (HER-SYS) has been used for managing COVID-19 cases since the end of May 2020. HER-SYS has 2 different versions. First, the digital system enables medical institutions to electronically report cases to the local health center [9]. The same system under a slightly different name, that is, MY HER-SYS, has functions that allow individuals who are quarantined to report simple self-health checkup information.

In addition, another app called COVID-19 Contact Confirming App (COCOA) increases the efficiency of containing and managing SARS-CoV-2 infection. This app enables users to use the Bluetooth functions on their mobile phones to receive anonymous notifications about the possibility of contact with a person who has been tested positive for COVID-19 [13]. When an app user inputs a randomly assigned processing number and the symptom onset date or test date on the app, the notification server verifies this processing number as the number issued to a positive tester [14]. The notification server in the management system operated by the Ministry of Health, Labor, and Welfare manages an app-generated daily key (an identifier unique to the device that changes on a daily basis), which can be provided to other app users. When the notification server verifies a processing number issued to a positive tester, the daily key is automatically provided to other app users via the notification server. The server does not store patients’ personal information or infection status, and informed consent is required to share their status with those who have been in close contact [15]. To further ensure privacy, the tracking system in the app is automatically disabled 14 days after requiring user’s approval [16]. The app also ensures that the information is only available for the user and allows the user to delete the record by deleting the app.

Japan’s quarantine measures for border protection, including on-arrival test, self-quarantine, or quarantine at designated accommodation, are no longer enforced since October 11, 2022 [17]. Before this date, those who arrived from foreign countries were required to use the app called MY SOS, a medical assistance and quarantine management app, when undergoing self-quarantine. The app allows the tracking of locations based on the GPS function; thus, the location of those who were under self-quarantine could be monitored by comparing their current location with the reported quarantine location [18]. It sends push notifications for video calls to confirm that the user is at the quarantine location and to require them to report basic health conditions daily [19].

Another app is the COVID-19 Vaccination Certificate App, which promotes efficiency when presenting vaccination records. When users install the app and scan their My Number Card (a version of social security card in Japan) using the near-field communication function on the mobile device, their vaccination record is automatically updated [20].

### Implications for Japan

In using the aforementioned technologies for the COVID-19 pandemic, Japan has faced several criticisms. The first and major criticism is the inefficiency of COVID-19–related technologies. Some processes, which include a combination of digital processing, manual checking, and writing of data by humans, have become the source of redundancy and inaccuracy. The use of the apps often involves coordination with the local government or the local health center, thereby impeding the optimal use of the apps and threatening data accuracy; the local health center employees are often required to manually input the faxed data with approximately 120 items for each COVID-19–positive case [21].

For example, a health center employee of Minato Prefecture in Tokyo pointed out that many problems have ensued from the use of HER-SYS; some of these problems are the inability to identify the number of individuals who are infected after transferring the data beyond the prefectural level and delays in contacting individuals with infection who tested in various ways [10]. The employee argued that the errors in the information were a serious issue. Given that HER-SYS could not include personal identification information, including name, address, and birth date, problems such as multiple filings of the same person by different prefectures were unavoidable and could only be prevented by relying on the local health center employees’ manual correction. Criticizing the discrepancy among the app’s capability, the necessary information, and the involvement of the health center and local governments, the employee evaluated that “HER-SYS is a system that was created by those who wanted to use the data, as its structure only focuses on collecting data without considering its actual operation” [10].

Frequent technical problems also hinder the full use of the app. For the first few months after introducing the COVID-19 Vaccination Certificate App, errors such as the failure of the near-field communication function to accurately read the users’ information and the filing of approximately 100,000 errors in data were reported [22]. For COCOA, technical errors that prevented the app from functioning on Android-based smartphones lasted for months without being fixed, affecting approximately 30% of all users [23]. The Ministry of Health, Labor, and Welfare of Japan reasoned that the problems were caused by the lack of awareness about the importance of running tests for its functions and the lack of proper personnel with IT knowledge [24].

Criticisms about other aspects of technological efficiency have also been raised. Given that the COVID-19 Vaccination Certificate App does not allow automatic update of information, record processing by the local government could take days to weeks to reflect the recent vaccination record on the app [25,26]. When people had moved to a new prefecture after their second vaccination and received their third vaccination at their new home, the local government in the previous prefecture managed their first and second vaccination records, and the local government of their new home managed their third vaccination record [26]. In this case, when they encountered an error, they had to resolve it by contacting the local government of both prefectures. For the vaccination information to appear on the app, a local government employee needed to manually input the vaccination information on the government’s vaccination record system, resulting in frequent errors in the data and delays in certificate issuance [27].

According to Sogabe [3], a professor of law at Kyoto University, the Japanese government’s collection of information using the apps to manage COVID-19 is strictly observant of the country’s privacy laws, mainly the Act on the Protection of Personal Information, considering that the apps are based on the user’s voluntary participation and that they promote transparent disclosure about their operation. In addition, Nakamoto et al [15] evaluated that among all apps using centralized Bluetooth digital health frameworks, COCOA has superior privacy protection measures. However, Sogabe [3] noted that the public does not have faith in the government’s observance of the laws related to personal information protection. He pointed out the loss of efficiency resulting from individuals’ unsubstantiated fear of being under surveillance by the government.

The most invasive collection of personal information was made using an app designed for use by people who were entering the country. They were required to consent to GPS tracking of their location during the quarantine period. They were also required to sign a pledge agreeing that, in case of violation, Japanese nationals may have their names publicly disclosed, and foreign nationals may have their residence status revoked or face deportation, in addition to the public disclosure of names and nationalities [28].

Furthermore, relatively minor concerns about social exclusion and equality associated with app use have been reported. For example, Yamagishi [29], a medicine and community health researcher, inferred that HER-SYS could not properly identify an outbreak in nursing homes in a timely fashion. Reporting cases of infection in the primary health care setting, often including individuals at high risk (eg, older adults), depends on manual reporting by health care workers, who are already extremely busy in taking care of patients or older adults [29]. However, this problem arose from the general lack of technological efficiency, rather than systemic shortcomings that cause social inequality in technology use.

The evaluation demonstrated that the voluntary use of digital technology for COVID-19 management in Japan by the public resulted in minimal concerns about privacy violations, social exclusions, and inequality. Instead, concerns were focused on the issues of technological inefficiency; government’s lack of consideration of end users, such as local government workers and physicians; and redundancy of a back-and-forth process between digital and manual data processing.

### South Korea’s Use of Digital Technology During the COVID-19 Pandemic

South Korea has been actively using various apps for all aspects of COVID-19 management, particularly before Omicron became the dominant SARS-CoV-2 variant. For those who did not own mobile phones, the local government had offered to rent them for free [12]. The national government actively encouraged and even socially mandated the use of various COVID-19 management technologies by the public. The government also sought to make technology adoption and use easy for citizens by engaging in the most used social networking services and South Korea’s search engine apps, including KakaoTalk and Naver [30], for the administration of vaccines, issuance of vaccination certification, and monitoring of individuals who are infected and quarantined.

The Self-Quarantine Safety Protection App is an app that was once required by those who were quarantined, including those who tested positive for COVID-19 or were in close contact with those who had tested positive and those who had entered from foreign countries. Using the app, the users were required to report basic health conditions twice a day. The app allowed location tracing for people in quarantine; hence, if the location of the users’ phone was reported to be outside the designated quarantine area, a public official appointed to their case would be notified and check their whereabouts. The location is traced according to the GPS system, but GPS-based tracking does not function well in indoor environment because of both issues associated with multipass and signal weakening caused by indoor obstacles [31].

To overcome errors from GPS, Wi-Fi positioning system (WPS) was built in the Self-Quarantine Safety Protection App [32]. The WPS stores the Wi-Fi signal of the quarantine area, preventing the false alert that the app users are outside the quarantine area as long as they are within the Wi-Fi signal’s reach [32]. In addition, given that WPS can only be built in Android-based apps, the Ministry of the Interior and Safety of South Korea sought to develop an “algorithm to determine whether a person has left the designated quarantine area by developing technology that combines built in cellphone censors and GPS” [32].

Since February 2022, the requirement for self-quarantine and the use of the app for those who were entering South Korea from foreign countries has been suspended [33]. However, before the rule was suspended, the violation of quarantine measures could result in 1 year of imprisonment or fine of Korean Won 10 million (US $7448.73), pursuant to the Infectious Disease Prevention Act.

The use of contact tracing QR codes, which are available through commonly used search engines and social networking service apps in South Korea, was mandated to be used from the beginning of 2022 until it was suspended. Until February 18, 2022, those who were visiting public places, such as restaurants and malls, had to provide their QR codes for contact tracing [34].

Moreover, Corona Overcome (COOV) is a decentralized identifier-based vaccine verification system app used in South Korea. In addition to allowing the users to present their vaccination information, the app enables cross identification, that is, users can provide vaccination verification information for each other [35]. The users can also control the type of personal information they want to disclose along with the vaccination verification. Since mid-December 2021, those who were entering public places were required to present the “vaccine pass” using COOV or other commonly used messenger and search engine apps in South Korea. Unvaccinated individuals could still enter some public places, but they had limited access to certain activities, including dining with others in a restaurant or entering a theater. In January 2022 and early February 2022, the government sought to expand the vaccine pass scheme, but the requirement was suspended because courts in some regions had ruled against it and issued temporary injunctions for disproportionately violating unvaccinated individuals’ basic rights, including the freedom to engage in daily activities and the right to pursue happiness [4]. In addition to the injunctions, the sharp rise in the number of Omicron cases increased the need to focus health care resources on the population that is infected. Eventually, the requirement was suspended as of February 2022 [36].

Other attempts to use technology on all fronts of COVID-19 management have been made in South Korea. Before contact tracing was suspended, Bucheon Prefecture proposed a pilot test of contact tracing using artificial intelligence (AI) [37]. The system allowed the use of AI algorithms and facial recognition technology to analyze footage gathered by closed-circuit television cameras around the city and to track the movements of a person who is infected. The AI-based system did not come to actualization because of privacy concerns, and it raised the question of how far the government can go in using technology for contact tracing for infectious disease management [37]. These social or legal requirements for technology implementation were aggressive compared with those in Japan, and accordingly, contrasting social implications were observed.

### Implications for South Korea

In South Korea, the government actively used high-end technologies, such as blockchain, to manage COVID-19 cases. Technology use led to more straightforward and rapid contact tracing and a relatively streamlined public implementation of infection prevention measures. The government continued to update the COVID-19 management technologies used in the apps [32]. Updates were necessary for the government to strongly convince the public to use the app and to efficiently implement relatively serious penalty for anyone who violated the quarantine measures under the Infectious Disease Prevention Act. However, the implementation of such technologies, which often required public participation, led to several social criticisms; for instance, technology use reportedly resulted in social exclusion, leaving out those who were socially disadvantaged or not tech-savvy.

Around the summer of 2021 (when vaccine demand was high but the supply was limited), vaccines were first provided to people aged ≥60 years and other vulnerable groups. However, when people would not show up to their reservations, the government made the app-based same-day reservation system available for other people willing to be vaccinated to prevent the waste of leftover vaccines [38]. This convenient system allowed people to find real-time information about which hospital had leftover vaccines and make vaccination reservations, even if they were not in the prioritized group. However, the vaccines were rapidly consumed, with many people reloading the app constantly to find an opening [5]. Usually, the remaining vaccines were supplied to only those who were young and tech-savvy or those with family members who could help them [39]. The Korea Medical Association issued a press release expressing concern over the decision to use the app as a way of allowing people to reserve leftover vaccines, because this app burdened the already exhausted health professionals and raised serious equality issues regarding vaccine distribution [40].

The policy that required the citizens to use the app daily as they visited public places, such as restaurants, for contact tracing and vaccination verification has also caused a problem. This policy was criticized because those without access to mobile devices were excluded from social activities [41]. Nevertheless, individuals without smartphones could fulfill the requirement for contact tracing and vaccine certification by writing down information for contact tracing and presenting the government-issued sticker for vaccination verification [42]. However, even with these alternative options, the active use of digital technology for COVID-19 management made the app’s use a de facto social requirement, making social activities inconvenient for those who could not use the apps. The policy also resulted in a “digital divide,” causing social isolation for those who could not navigate the COVID-19 management apps [6].

Broadly requiring contact tracing was a “striking exception to Korea’s stringent legal regime for data protection,” especially in that the tracing system was centralized, with the government’s Epidemic Investigation Support System organizing and providing locational information for epidemiological investigations [43]. Although this centralized system allowed timely, streamlined, and effective epidemiological investigation, many countries, including Japan, preferred a decentralized Bluetooth-based system or a partially centralized system to address privacy concerns [43]. The adoption of the centralized system for contact tracing may have been enabled as the levels of public support and willingness to cooperate with an effective infectious disease control system were fairly high [43]. As the previously mentioned explanation by Sogabe [3] suggests, given that the Japanese citizens were not willing to entrust their personal information to the government, the governments of Japan and South Korea may have opted to use the most suitable technologies while considering their public’s sentiment about privacy protection.

Nonetheless, in South Korea, the mandated use of technologies has raised serious concerns about the potential invasion of privacy and the right to self-determination regarding the release of personal information and medical decisions. This report is ironic, considering that the contact tracing system using QR codes has been established with the purpose of protecting personal privacy. Initially, when the visitors at public places were asked to write down their names and cellphone numbers for contact tracing, cases of privacy violation, personal information mishandling, and falsification were reported [44]. To solve this problem, the government started to use a QR code–based system. However, despite the government’s intention, with the daily use of the contact tracing app coupled with the vaccine certification system, many people expressed anxiety about the government obtaining and using their personal information, such as their whereabouts [45].

While adopting the centralized system, the Korean government sought to protect privacy by using a bifurcated system, which separates personal data from QR codes [43]. When people entered a public venue and scanned the “ephemeral and pseudonymized QR code,” the data containing no personal information were forwarded to the Social Security Information Service, whereas encrypted personal information was separately stored by mobile platforms or app development companies [43]. The data from the scanned QR code were only matched with the personal information if the visitor was confirmed to be positive for COVID-19; then, relevant data were sent to the Epidemic Investigation Support System [43]. Therefore, although maintaining the centralized system that allowed effective epidemiological investigation, the Korean government sought to protect private information by adopting the bifurcated system. Nonetheless, information about personal data and the locations visited by the app users was still disclosed to the government once they tested positive for COVID-19, with no request for consent from them. In addition, technological measures to ensure data protection, although important, insufficiently alleviated the criticism of invasion of personal privacy by South Korea’s contact tracing system.

On evaluating South Korea’s use of COVID-19 management technologies, Park et al [46] explained that although the urgency for disease control during the COVID-19 pandemic may justify the government’s collection and sharing of personal data from its citizens, proper protection of personal privacy by the current system cannot be assured. With this observation, they emphasized that when this pandemic is over, social discussions about how to develop a system that can allow optimal collection of data while offering proper protection for privacy should be conducted.

In summary, Table 2 illustrates the characteristics of digital technology implementation for COVID-19 management in Japan and South Korea and the contrasting social implications from the implementation.

**Table 2:** Characteristics of digital technology used for COVID-19 management and social implications for Japan and South Korea.

Country	Characteristics of digital technology used for COVID-19 management	Social implications resulting from technology use		
		Inefficiency of technology use	Concerns related to the violation of basic rights	Infringement of social values
Japan	Participation (the use of the apps) was voluntary (except for those who have entered from foreign countries) Frequent or long-lasting technical difficulties	Inefficiency was criticized, especially regarding the usability of the apps, and the reporting system consisted of redundant manual processes	Limited—because users with privacy concerns can choose not to use the apps	Limited concerns related to the reporting system for those in primary care settings
South Korea	Participation (the use of the apps) was largely required either socially or legally Comparatively short and few instances of technical difficulties	Limited concerns during the short instances of technical difficulties	Privacy concerns regarding the release of personal information for the use of the apps Injunctions were issued based on potential concerns about the violation of the freedom to engage in daily activities and the right to pursue happiness	Serious concerns related to inequality for older adults and those who could not appropriately use the technology

## Discussion

### Limitations

The limitation of this study lies in how the use of the abovementioned technologies has been affected by the differing government systems, privacy laws, and cultures of the 2 countries. In the future, comparative studies between numerous countries may shed more light on the social implications of using pandemic management technology. Nevertheless, this study demonstrates contrasting social implications resulting from 2 different approaches to the use of technology during the pandemic; one approach was marked by ineffective technological implementation, and the other approach was associated with concerns about equality and privacy infringement.

### Social Implications of Using Technology for COVID-19 Management

The most substantial differences in the use of pandemic management technology in Japan and Korea lie in the extent of its use and its mandated use. In Japan, the use of the apps was generally voluntary. Criticisms about the use of digital technology were centered on the lack of efficiency or inadequate use of technology. In South Korea, the use of most COVID-19 management apps was mandatory, either socially or legally, until February 2022, considering that using the apps was often the only feasible way to engage in social activities and abide by the quarantine rules. Users had limited freedom to control what kind of personal information they were willing to share and for which purposes. Therefore, there were serious concerns regarding potential infringement of privacy and equality, freedom to engage in daily activities, and the right to pursue happiness. In both countries, the most strictly imposed rules involved quarantine measures for travelers coming from a foreign country.

In South Korea, according to a survey conducted by the Pew Research Center, the widespread use of the app by the public may be explained by a high rate of cellphone ownership, with 95% of its citizens owning a cellphone in 2019 [47]. In addition, differences in the government systems may have contributed to the efficiency of technology use. Compared with Japan, where the personal information about residents is mainly handled by local governments, South Korea has a long-established centralized national social security system providing each citizen with a personal identification number, thereby making demographic data and health record management inherently more organized for the government. Hence, a centralized contact tracing system can be seamlessly implemented.

Active collection of personal information using QR codes allowed the Korean government to efficiently track individuals who are infected and quarantined, but it also highlighted concerns about potential invasion of privacy. Corona 100m, an unofficial GPS-based contact tracing app released in South Korea by a company called Tina3d, has also emerged as a matter of concern for privacy, as app users could see personal information about those who have been infected (eg, date of infection, nationality, sex, age, and location visited) [48]. COCOA was developed by the Japanese government; thus, comparison between these 2 apps cannot be fully appreciated. However, the difference in the degree of privacy concerns caused by these 2 contact tracing apps with different privacy measures demonstrates how the structure and execution of an app can manage social concerns resulting from pandemic management technology use.

Although strongly “encouraging” the use of apps by the public, the South Korean government sought to enhance privacy protection by adopting the best available technology. For example, to protect user information and prevent the manipulation of vaccination information, COOV, which uses blockchain technology, was developed [35]. More specifically, by using a decentralized identifier, users were assured that data, including the issuer and owner’s identification, cannot be changed once recorded [35]. Therefore, although the government’s effort to use the best available technology may have been inadequate to protect personal information and privacy, the efforts allowed efficient use of pandemic technologies in South Korea. In particular, active collaboration with private technology companies enabled the government to quickly develop and use technology to manage COVID-19 cases.

In both countries, the COVID-19 pandemic has demonstrated the importance of the role of technology in effectively controlling infectious diseases. In addition, the contrasting implications from different approaches demonstrate that, while using pandemic management technology, balancing technology use efficiency and social values and human rights protection is important. These findings are supported by those of other studies. He et al [49] explained that technology use for pandemic management can raise “challenges such as security, privacy, biases, ethics, and the digital divide.” Furthermore, “digital interventions come at a price,” especially because the use of digital technology that uses personal data can “potentially threatens privacy, equality and fairness” [50]. Although requiring the use of the apps may increase efficiency while managing infectious diseases, app use may also result in many serious social issues and equity and privacy concerns. Therefore, Sowmiya et al [51] argues that, to address privacy concerns, it is important to protect users of contact tracing apps from mandated use or unwanted interference.

As demonstrated by Bucheon Prefecture’s attempt to use AI technologies for contact tracing [37], the more developed the pandemic management technology becomes in the future, the more pertinent the need to consider privacy becomes before implementing the technology. Countries, including Japan and South Korea, are left with lessons from their own past experiences to improve technology use for infectious disease management.

### Recommendations

The comparative analysis and discussions in this study have yielded 3 general recommendations for using technology for infectious disease management.

First, public education about the technology and the extent of collection and use of private information by apps can help gain full cooperation from the public. Although the Korea Center for Disease Control and Prevention and Blockchain Labs, developers of COOV, have also used blockchain technology to promote personal privacy and ensure the security of the vaccine certification app, many Koreans are still unaware of how well their privacy is protected and how secure the app is. Therefore, the government, by undertaking more efforts to disseminate information to the public about how private information is properly managed and controlled, could have partially alleviated the public’s concerns. In the web-based survey analysis by Oh and Suh [52] about the disclosure of personal information in 594 COVID-19 cases, individuals were more adherent if they believed that disclosing personal information has a positive impact on COVID-19 management; thus, promoting public awareness about the positive impact of personal information disclosure is necessary.

In Japan, the importance of educating the public has also been stressed. A study found that educating the public about the app, including its benefits and its privacy protection, is integral to promoting its widespread use, such as COCOA [53]. Abuhammad et al [54] suggested that developing well-organized and easy-to-understand guidelines regarding data gathering and the use of contact tracing technology is crucial. Providing the public with such information in a comprehensible way can be effective both for promoting the public’s faith about privacy protection in the use of COVID-19 management technology and for promoting more active use of the technology by the public.

Second, before introducing the technology for infectious disease control to the public, the government needs to prepare for the possibility of technological malfunctioning, especially if the technology is intended to be widely used by the public. Both the Japanese and South Korean governments had to experience trials and errors in developing national pandemic management technology, including technical difficulties. In Japan, technical errors in apps, such as COCOA, have prevented effective use of the technology for COVID-19 management for several months. The malfunctioning of the apps was less frequently reported and more quickly resolved in South Korea. However, considering that the public was required to use the apps, such as COOV and the contact tracing app, the consequences of any app malfunctioning were considerably grave. On December 13, 2021 (the first day of mandating proof of vaccination for certain public activities), some users reported app malfunctioning, causing considerable panic and confusion [55]. Those who have an obligation to require vaccination proof, such as restaurant owners, had not been instructed about what to do in case the app malfunctioned. Owing to fear of potential penalties, some business owners could not allow guests to enter their places of business if their app malfunctioned [55]. Finally, the Korea Center for Disease Control and Prevention announced the postponement of enforcing this mandate until the next day, and all issues were resolved within the day. However, the users whose app malfunctioned had already had their daily activities disrupted. Hence, when a government uses a digital vaccination certification system for vaccine mandates, it should be aware of the potentially grave social consequences in case the app malfunctions. Therefore, for the widespread use of infectious disease prevention technology, the public should be provided with basic instructions about what they need to do when the technology malfunctions.

Finally, for some technologies, such as the quarantine management app, to be truly effective, people under quarantine must be required to use this app. However, the more socially or legally required the use of technology is for the public, the more rigorous the consideration of human rights, including privacy and equality, should be made. For example, in South Korea, some business owners complained that the strict requirement for all sports clubs, including those providing a venue only for low-intensity physical activities, was unfair, considering that unvaccinated individuals could relatively freely still use other facilities. [56]. Furthermore, when the presentation of a digital vaccine pass was required to enter public facilities, older adults who had access to neither the app nor alternative ways of proving vaccination experienced substantial inconvenience [57]. Therefore, while promoting the use of an app for pandemic management, careful examination about potential social issues that can result from the use must be made.

Before mandating the use of technology for infectious disease control, the government needs to first weigh up and resolve questions such as the following: to what extent can individual autonomy be sacrificed for the effective control of an infectious disease; does technology use achieve public consensus; and which measures can be taken to ensure protection of privacy, equality, and other human rights? As in the case of South Korea and other countries, including the United States, the judicial system can ensure that the government’s infectious disease control measures do not overly violate the rights of the public.

The Japanese and South Korean governments are already taking measures to improve their systems. In Japan, to enable effective use of technology, a governmental agency called “Digital Agency” has been established. By recognizing technical shortcomings during COVID-19 management, the agency reported a plan to promote effective technology use, including the development of a new infectious disease control system called “Subsequent Infectious Disease Control Surveillance System,” collaboration with private companies, and the promotion of the active use of “My Number Card” [26]. In South Korea, measures have been taken to ensure proper privacy protection and optimal pandemic management technology use in the future; these measures include responding to public concerns and opinions regarding pandemic management technology use, facilitating technology development that can optimize data protection, and calling for public participation by seeking recommendations regarding technological solutions during the COVID-19 pandemic [58-60].

### Conclusions

The comparative analysis of digital technology use in South Korea and Japan highlights matters that need to be considered for the future use of technology for infectious disease management. In promoting the use of technology to effectively manage a pandemic and facilitate social and economic activities while controlling the spread of infection, the government and the public should have ample social discussions before the adoption of technology to reach a social consensus regarding the necessity and extent of its use. To alleviate the public’s fear about privacy breach when using an app, the government should educate the public regarding the functions of the app, the technology it uses, its benefits, personal information to be used, and the way this information will be used. If technology use is required for the citizens, questions about equality, fairness, and privacy should be considered. Those who cannot effectively use the technology should be given sufficient consideration, including providing alternative ways to meet the same requirements. Measures for when a widely used infectious disease control technology malfunctions should be established in advance to prevent social chaos.

The COVID-19 pandemic has taken countless lives and tremendously disrupted the public’s way of living, but it leaves important lessons regarding the use of technology for infectious disease management in the future. Notably, although technology use can increase efficiency in managing infectious diseases, it can also result in many social issues and violation of rights. However, when it is used with utmost care and consideration, it can be greatly beneficial, with limited problems. Thus, before the next possible pandemic, the government and the public must discuss about what the society prioritizes during the time of pandemic and what they should achieve with the use of technology. In addition, the government should actively collaborate with and support private technology companies to develop more efficient pandemic management technology with enhanced data protection.

## Abbreviations

AI: artificial intelligence
COCOA: COVID-19 Contact Confirming App
COOV: Corona Overcome
HER-SYS: Health Center Real-time Information-Sharing System on COVID-19
WPS: Wi-Fi positioning system
